# Characterization of cyanobacterial isolates from freshwater and saline subtropical desert lakes

**DOI:** 10.1007/s12223-022-01016-w

**Published:** 2022-12-12

**Authors:** Ehab Shawer, Hosam Elsaied, Ahmed El-Gamal, Shawky Sabae

**Affiliations:** 1 Department of Microbiology, National Institute of Oceanography and Fisheries, NIOF, Cairo, Egypt; 2 Department of Genetics and Genetic Engineering, National Institute of Oceanography and Fisheries, NIOF, Cairo, Egypt; 3 Botany and Microbiology Department, Faculty of Science (Boys), Al-Azhar University, Nasr City, Cairo, 11884 Egypt

**Keywords:** Cyanobacterial isolates, Desert lakes, Molecular characterization, Morphology, Biochemical contents

## Abstract

Characterization of Cyanobacteria in lakes with different physicochemical properties provides insights into the diversity of this phylum and knowledge of their features that are relevant to biotechnology applications. Six Cyanobacterial isolates were recovered from freshwater Lake Nasser and saline Lake Qarun, Egypt. The isolates were identified based on both morphology and molecular markers, 16S rRNA, and RuBisCO *cbbL* genes. The isolates SN1, SN2, SN3, SN4, Q1, and Q2 showed homologies with *Merismopedia*, *Oscillatoria*, *Limnothrix*, *Persinema*, and *Jacksonvillea*, respectively. The *cbbL* sequences for isolates SN1, Q1, and Q2 represented the first records for candidates relating to the genera *Merismopedia* and *Persinema*, and *Jacksonvillea*, respectively. Biochemical contents, carbohydrates, proteins, lipids, pigments, and ash-free dry weight were measured for each isolate. Isolate SN2 had the highest content of allophycocyanin, 71 ± 4.8 mg/g DW, and phycoerythrin, 98 ± 6.7 mg/g DW, while the isolate SN4 had the highest composition of total protein, lipid, carotenoid, and chlorophyll a, recording 364.7 ± 6.4 mg/g DW, 67.6 ± 0.2 mg/g DW, 0.261 ± 0.01 mg/g DW, and 10 ± 0.6 mg/g DW, respectively. Isolate Q1 recorded the maximum amount of phycocyanin, 114 ± 20.7 mg/g DW among isolates. The isolate Q2 was observed to have the highest carbohydrate content, 274 ± 14.5 (mg/g DW), and ash-free dry weight, 891.8 ± 2.8 mg/g DW. Thus, the study indicated that the current isolates may represent promising resources for biotechnological applications.

## Introduction

Cyanobacteria are geographically widespread autotrophs, inhabiting a wide range of environments, and constituting the main primary producers in many aquatic ecosystems (Whitton and Potts 2000). Due to the continuous uncovering of cryptic Cyanobacterial species, taxonomic identification of Cyanobacteria has become a challenge for phycologists. The first trials for the taxonomy of Cyanobacteria were done in the late nineteenth century. Rippka et al. (1979) recorded five orders of Cyanobacteria. Order I represented Chroococcales, which includes single-celled Cyanobacteria, multiply by binary fission and present solitary or in colonial shape. Order II, Pleurocapsales, constitutes coccoid Cyanobacteria, which are characterized by the formation of baeocytes. Order III, Oscillatoriales, includes filamentous Cyanobacteria formed only from vegetative cells. Order IV, Nostocales, composes of filamentous taxa capable of producing heterocytes and akinetes. Order V, Stigonematales, includes filamentous taxa which also form heterocytes and akinetes and are characterized by their ability to form true-branched trichomes (Castenholz et al. 2001). However, Rippka’s taxonomic scheme for Cyanobacteria, based on morphological traits, e.g., multicellularity, baeocyte formation, presence of akinetes, tapering, polarity, and branching patterns, has left overlapped phyletic groups, and consequently, does not always accurately represent evolutionary relationships.

As molecular identification tools become available, taxonomic updating for Cyanobacteria has been required (Komárek et al. 2014; Dvorak et al. 2015). With the introduction of the rRNA gene as a molecular taxonomy tool for the identification of Cyanobacteria, more than 92000 rRNA gene sequences have been deposited in DNA databases, http://ddbj.nig.ac.jp/arsa/search?lang=en&cond=quick_search&query=16S+Cyanobacteria&operator=AND, characterizing various taxonomic ranks of Cyanobacteria (Walter et al. 2017). Few studies have applied genetic markers to describe the taxonomy of uncultured Cyanobacteria in African lakes. Elsaied (2007) used 16S rRNA gene metabarcoding for recording 10 uncultured cyanobacterial phylotypes from Lake Manzala, Egypt. Uncultured Cyanobacteria-like 16S rRNA gene phylotypes have been recorded in desert lakes of Wadi An Natrun, Egypt (Mesbah et al. 2007). Dadheech et al. (2009) have used PCR-based denaturing gradient gel electrophoresis (DGGE) for 16S rRNA gene to identify uncultured Cyanobacteria in marine alkaline and freshwater lakes of Kenya. *Arthrospira platensis* NIOF17/003 has been isolated from El-Khadra saline-alkaline lake, Wadi El-Natrun, Egypt, characterized as a novel species, based on 16S rRNA gene barcoding, and screened for biodiesel metabolic production (Zaki et al. 2021).

However, the 16S rDNA has recently been shown to lack the power of characterizing lower taxonomic ranks, such as species and strains (Konstantinidis et al. 2006; Goris et al. 2007). The level of the 16S rRNA gene nucleotide identity that has been accepted to characterize prokaryotic species identification has been calculated as 98.65% (Kim et al. 2014). There have been records for differentiated populations of phenotypically different Cyanobacteria that had identical 16S rRNA gene sequences, though they varied considerably in metabolic features (Miller et al. 2006).

So, other potential molecular markers have been added for enhancing clarification of Cyanobacterial taxonomy. The gene *cbbL*, which encodes the large subunit of ribulose 1,5, bisphosphate carboxylase oxygenase, RuBisCO, the key enzyme of the Calvin cycle, has been considered as an efficient molecular marker for characterizing the functional phylogeny of Cyanobacteria (Dvorak et al. 2014). However, *cbbL*-based taxonomy has been used for the classification of more than 60 species of Cyanobacteria, http://ddbj.nig.ac.jp/arsa/search?lang=en&cond=quick_search&query=cbbl+Cyanobacteria&operator=AND. Hence, the combination of the 16S rRNA gene data with those of other molecular markers, collectively known as multilocus sequence analysis (MLSA), has become common in molecular studies to address the taxonomic gaps of the 16S rRNA gene, giving almost robust phylogenetic characterization (Wilmotte et al. 2017). A novel species, *Dolichospermum hangangense* has been taxonomically characterized by using MLSA of the 16S rRNA gene and *cbbL* (Choi et al. 2018).

Lake Nasser is one of the largest worldwide artificial freshwater reservoirs, (Imam et al. 2020). According to current speed, turbidity, nutrient availability, and suspended solids, the lake has been divided into three sections: riverine, transition zone, and lacustrine section, where the first two sections are eutrophic, while the third section is mesotrophic. Cyanobacteria have been recorded as the abundant group in the transition and lacustrine sections (Salem 2011; Abdel-Gawad and Abdel-Aal 2018). Cyanobacterial genera, such as *Anabaena*, *Aphanizomenon*, *Chroococcus*, *Lyngbya*, *Merismopedia*, *Microcystis*, *Nostoc*, *Oscillatoria*, *Phormidium*, and *Spirulina*, have been microscopically observed at Lake Nasser. *Synechococcus*- and *Oscillatoria*-like 16S rRNA gene phylotypes have been recorded in the guts of Nile tilapia, *Oreochromis niloticus*, along Lake Nasser (Elsaied et al. 2019).

Lake Qarun has been considered a saline eutrophic lake, located as a depression in the western desert of Egypt. It is an inland closed lake that receives about 450 million m^3^/year of agricultural runoff from drains, El-Bats, and El Wadi (Redwan and Elhaddad 2017). Salinity in Lake Qarun varies between 14.24 and 39.8 g/l (Abd El-Aal et al. 2020). The main phytoplankton fractions in Lake Qarun have been found to belong to diatoms, dinoflagellates, and Cyanobacteria (Abd El-Karim 2012; Zaher and Ibrahim 2018). Cyanobacterial fraction constituted about 26.8% of the total phytoplankton cell concentration in Lake Qarun (Flefil and Mahmoud 2021). The microscopic survey has observed Cyanobacterial genera *Anabaena*, *Chroococcus*, *Gomphosphaeria*, *Merismopedia*, *Microcystis*, *Oscillatoria*, and *Phormidium* at the lake (Fathi and Flower 2005).

Cyanobacteria produce various types of natural products, considering this phylum as one of the main aquatic bioresources for several biotechnological applications (Singh et al. 2018). Cyanobacteria are important sources of carbohydrates, proteins, lipids, natural pigments, and novel bioactive compounds, such as oscillapeptin A, hapalindole A, alkaloids, lipopeptides, fatty acids, anabaenopeptin E, sester-terpenes, lyngbic acid, glycolipids, macrolactones, glycosidic macrolides, and tetrasaccharides (Nagarajan et al. 2012; Rai and Rajashekhar 2015; Demay et al. 2019). Some studies have shown the relationship between trophic status and the metabolic potential of Cyanobacteria in freshwater lakes (Shen et al. 2019).

The studies on cyanobacterial diversity in Egyptian lakes have been limited to conventional taxonomic methods, based on morphology, and some traditional biochemical analyses. The current study explored and characterized some novel freshwater and saline cyanobacterial species from Egyptian lakes, based on morphology and two molecular tools, 16S rRNA gene coupling with *cbbL* barcoding. The biochemical composition of the isolates was determined to assess their biotechnological potential.

## Materials and methods

### Sampling

**Fig. 1:**
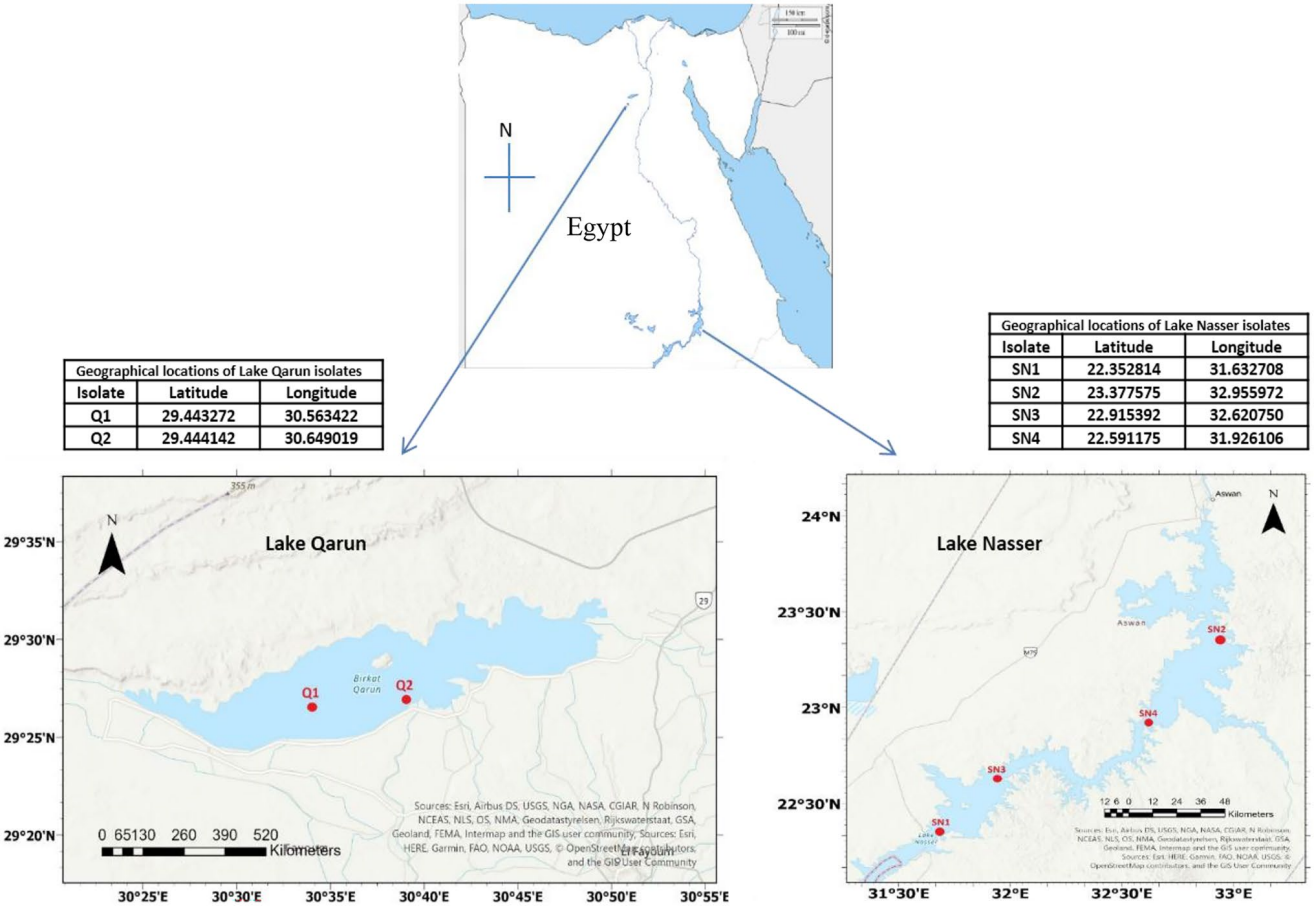
Map shows the isolate sampling sites in Egyptian lakes, Lake Qarun, and Lake Nasser

Sampling sites from Lake Nasser and Lake Qarun are shown in Fig. 1. Water samples, each of 500 mL, were collected in sterilized bottles and maintained at low-temperature conditions (2–8 °C) for culture and isolation procedures. Ten milliliter of water samples was inoculated into 250-mL Erlenmeyer flasks, which contained 100 mL of BG11 medium (Allen 1968). The pH of the culture medium was adjusted to 7.1. For saline cultures, NaCl (10 g/L) was added to prepare marine BG11 medium. All inoculated flasks were incubated at 25 °C with a 16:8 light–dark cycle of 37 µmol. m^−2^ s^−1^ photon flux density until reaching stationary phase (3–4 weeks).

### Isolation and identification of Cyanobacteria

Cyanobacteria were isolated and purified through serial dilutions, optical microscopic examination, picking with Pasteur micropipette, spreading on an agar plate, and frequent sub-culturing for obtaining the pure isolates (Guillard 2005; Andersen and Kawachi 2005).

### Morphology

The morphological features, including cell dimension, cell shape, presence or absence of calyptra, heterocysts, akinetes, and sheath characters, were described for each isolate, using an inverted microscope, Zeiss Axiovert 25, Carl Zeiss. Species characterizations were carried out based on classical algae keys (Kützing 1845; Gomont 1892; Meffert 1988; Castenholz 1989; Komárek and Anagnostidis 2005), concerning algae database (Guiry and Guiry 2021) http://www.algaebase.org/).

### Molecular characterization

Genomic DNA was extracted from 2 mL of each Cyanobacterial pure isolate culture, using the DNeasy Power Water kit (Qiagen, catalog no.14900–50-NF), with modifications of Elsaied et al. (2002). The culture sample was centrifuged, and the cell pellets were washed several times with TE buffer, 10 mmol/L Tris, 1 mmol/L EDTA, pH 8. The algal cells were lysed with glass beads and lysis buffer in the extraction kit. The purified DNA was run on 0.9% agarose gel, stained with ethidium bromide, and visualized by Gel Doc™ XR + imager (Bio-Rad, UK, Catalog number 1708195). The extracted DNA was kept at − 20 °C for further molecular analyses.

PCR for amplification of the 16S rRNA gene was performed using primers, EuBac-27F, 5′-agagtttgatcctggctcag-3′, and EuBac-1492R, 5′-ggttaccttgttacgactt-3′ (Lane 1991), producing amplicon with size ~ 1500 bp. The primers IAB-595F, 5′-gayttmactaargatgayga-3′, and IAB-1385R, 5′-tcgaacttgatttctttcca-3′ (Elsaied and Naganuma 2001), were used to amplify 800 bp of the RuBisCO large subunit-encoding gene, *cbbL*. PCR was performed in 50 μL volume of reaction mixture, 5 μL MgCl_2_ (2.5 mM), 5 μL dNTPs (2.5 mmol), 5 μL EX taq buffer (Mg^2+^ free) 10 × , 2 μL of each primer (10 pmol), 0.3 μL Takara EX-Taq™ Polymerase 250 U, and 2 μL DNA template (25 ng). PCR was carried out by a ProFlex™ thermal cycler (Life Technology, USA). The PCR program for amplification of the 16S rRNA gene included an initial denaturation step at 95 °C for 3 min, followed by 30 cycles, each one consisting of 50 s at 95 °C, 50 s at 55 °C, and 1 min at 72 °C, with a final extension step at 72 °C for 12 min. For amplification of the gene *cbbL*, thermal cycling was initiated with denaturation at 95 °C for 1 min, followed by 30 cycles of denaturation at 95 °C for 50 s, annealing at 49 °C for 1 min, and extension at 72 °C for 2 min, followed by final extension step at 72 °C for 12 min. The PCR products were purified using a QIAquick^®^ PCR purification kit (Catalog no. 28104, Qiagen, Germany) and sequenced using a Sanger ABI 3730xl capillary DNA analyzer (Applied Biosystems, Foster City, CA, USA).

### Phylogenetic analysis

Sequences were submitted to the BLASTN homology search tool, for screening their similarities with those deposited in the DNA database. The nucleotide sequences of the gene *cbbL* were translated into deduced amino acids, using the Transeq tool, https://www.ebi.ac.uk/Tools/st/, and submitted to the BLASTP homology search tool. Phylogenetic trees, based on partial nucleotide sequences, for the 16S rRNA gene, and deduced amino acid sequences, for *cbbL*, were constructed through two analyses. First, recorded sequences were aligned with those from related members of Cyanobacteria deposited at databases, using Clustal Omega, http://www.ebi.ac.uk/Tools/msa/clustalo/. Second, consensus trees were drawn through MEGA11 software (Tamura et al. 2021), using compatibility of phylogeny algorithms, maximum likelihood, neighbor-joining, and maximum parsimony, with the bootstrap confidence level, 1000 replicates. The 16S rRNA gene sequences of current isolates were deposited in the gene bank under accession numbers from MZ504747 to MZ504752, while *cbbL* sequences were recorded under accession numbers from MZ702731 to MZ702736.

### Phytochemical screening

#### Total carbohydrate

Total carbohydrate content was determined using the phenol–sulfuric acid method with modification of Quero-Jiménez et al. (2019). The absorbance of the extract was measured at 490 nm, and the total carbohydrate content was calculated from a linear regression equation obtained from the glucose standard curve.

#### Total protein

The total protein content was determined using the Folin–Ciocalteau reagent (Lowry et al. 1951). The extract absorbance was measured at 660 nm, and the sample’s protein content was calculated from a linear regression equation obtained from the bovine serum albumin standard curve.

#### Total lipid

The total lipid content was determined by the gravimetric method (Folch et al. 1957), in which 0.5 g of the dry weighted sample was extracted with a solvent mixture of methanol-chloroform (1:2 v/v), and the filtrate was collected in a pre-weighed flask and evaporated, and weight of dry material was determined. The difference between the initial weight and final weight gave the total lipid content.

#### Chlorophyll-a determination

Chlorophyll-A was determined according to Lorenzen (1967). About 4 mL of Cyanobacterial culture was centrifuged, and the pellets were homogenized, followed by chlorophyll-a extraction with 90% aqueous acetone. Chlorophyll-a absorbance was measured at 750 nm and 665 nm, before and after acidification with two drops of 1 mol/L HCl for correction against phaeopigments. The chlorophyll-a was calculated using the following equation.

$$\mathrm{Chl-a}\ \mathrm{(\mu g/L)}=26.7\mathrm{[(A665^b-A750^b)-(A665^a-A750^a)]}\ \mathrm{v/VI}$$

$$\mathrm{Chl-a}\;{(\mathrm {mg/g}\;\mathrm D\mathrm W)}=\text{Chl-a}\ \mathrm{(\mu g/L)}-1000 \times\mathrm{Y}$$

- A665^b^ -A750^b^ = Absorbance at 665 and 750 before acidification.
- A665^a^-A750^a^ = Absorbance at 665 and 750 of the acidified extract.
- *v* = volume of the extract (mL).
- *V* = volume of the filtrate (L).
- *I* = light path of the cuvette (cm).
- *Y* = Dry weight yield in grams

#### Phycobiliprotein content measurements

Phycobiliproteins were extracted through centrifugation of 250 mL of stationary phase growing culture, at 4000 rpm for 10 min, and the supernatant was decanted. The cell pellets were suspended in 10 mL of 0.1 M phosphate buffer pH 7.0, homogenized, and extracted by sequential freezing and thawing cycles until the pellets turned greenish. The homogenate was centrifuged at 4000 rpm for 10 min, and the phycobiliproteins-containing supernatant was collected into a clean tube. The phycobiliproteins were measured with a spectrophotometer, under absorbance at 280 nm, and 562 nm for phycoerythrin; 615 nm for phycocyanin; and 652 nm for allophycocyanin, followed by quantifications using equations of Bennett and Bogorad (1973).

- Phycocyanin concentration: 
  $$\mathrm{(PC) mg/mL = [A615-0.474 (A652)]/5.34}$$
- Allophycocyanin concentration: 
  $$\mathrm{(APC) mg/mL} = [\mathrm{A}652-0.208 (\mathrm{A}615)]/5.09$$
- Phycoerythrin concentration:
  $$\mathrm{(PE) mg/mL} = [\mathrm{A}562-2.41\mathrm{(PC)}-0.849 \mathrm{(APC)}]/9.62$$

All phycobiliproteins were normalized to mg/g DW by dividing their content in liters by the corresponding dry weight yield in grams.

### Determination of carotenoids

Carotenoids were determined based on the methodology of Zavřel et al. (2015). One gram of dry weight biomass was extracted with absolute methanol and kept protected from light at 4 °C for 20 min, and the carotenoid-containing supernatant was measured under absorbance wavelengths of 470 nm, 665 nm, and 720 nm. The carotenoids were calculated through the following equations:

$$\mathrm{Chl a}\ \mathrm{(\mu g/L)}=12.9447\ \mathrm{(A665-A750)}$$ (Ritchie 2006)

$$\mathrm{Carotenoids}\ [\mu \mathrm{g}/\mathrm{mL}]=[1000\ (A470-A720)-2.86$$$$(\mathrm{Chl}\ \mathrm{a})]/211$$ (Wellburn 1994)

### Ash-free dry weight (AFDW) content

Ash-free dry weight content was determined according to AOAC (2000) protocol. About 0.5 g of the dry weight sample was incinerated in a furnace at 500–550 °C until the white ash was formed (about 5 h), and the dry weight of the ash was estimated.

$$\mathrm{AFDW}=\mathrm{total}\ \mathrm{dry}\ \mathrm{weight-ash}\ \mathrm{weight}$$ 

### Statistical analysis

Statistical analysis was carried out with XLSTAT 2019.1 software. All the results were calculated as mean ± standard deviation. One-way ANOVA was applied to test for significant differences at *p* < 0.05.

### Results

#### Characterization of the isolates

**Fig. 2:**
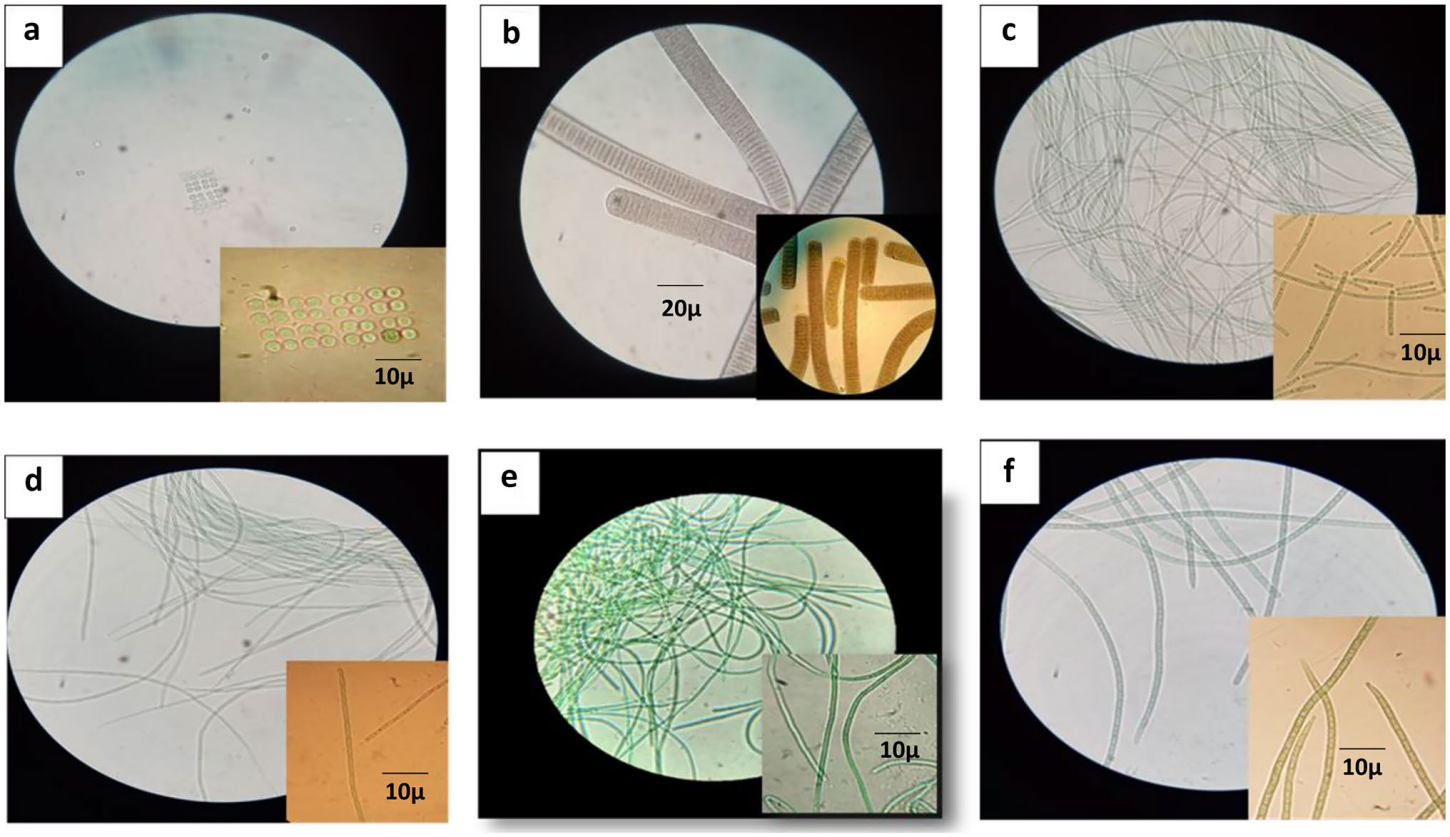
Photomicrographs show morphology of Cyanobacterial isolates. **a** Isolate SN1, **b** isolate SN2, **c** isolate SN3, **d** isolate SN4, **e** isolate Q1, **f** Isolate Q2

Six Cyanobacterial isolates, four from Lake Nasser and two from Lake Qarun, were recovered (Fig. 2). The freshwater isolate SN1, from Lake Nasser, (Fig. 2a), has a colonial form, with 4–16 up to 64 cells, per colony, arranging perpendicular in longitudinal and transverse rows, forming quadrangular colonies, which enclosed by a distinct, hyaline, and homogeneous mucilage. Cells are spherical or hemispherical, with 2.5–4.0 µm diameters and 2.0–3.0 µm in length, having bright blue-green and homogenous content.

The freshwater isolate SN2, from Lake Nasser (Fig. 2b), has trichomes, which form a brown mass on BG11 medium, straight not tapering toward the apex. The apical cell is rounded and slightly capitated with a thick outer membrane. Cells have 15 µm in width and 1.3–3.0 µm in length and granulated and distinctly constricted at the cross wall. The isolate has vegetative reproduction, which is carried out by the disintegration of trichomes into small fragments through the formation of necridic cells.

The Lake Nasser isolate SN3 (Fig. 2c) has a thin thallus, filament solitary, free-floating (planktonic), with slow motility by gliding with oscillation. Filaments are unsheathed, not constricted at the cross wall, and not attenuated at the apex. Trichome has a pale blue-green color and consists of narrow cylindrical, elongated cells, with 1.0–1.5 µm in width and 5.0–6.0 µm in length. The isolate contains obvious gas vesicles and polar aerotopes. Apical cells are cylindrical without calyptra.

Isolate SN4, Lake Nasser (Fig. 2d), has morphological characteristics similar to isolate SN3, but its growth differs slightly, as the trichrome is tangled, forming mats at the bottom of the culture vessels, and the cells contain less obvious gas vesicles.

Isolate Q1, Lake Qarun (Fig. 2e), has a filamentous form, solitary and tangled into clusters to form mats, being attached to the substrate. Trichomes are motile with thin, firm, and colorless sheaths, opening at the apex. Filaments are cylindrical, not constricted at the cross walls, not attenuated at the apex with rounded apical cells, and the end cells are without thickened walls or calyptras. Trichomes have a bright blue-green color and compose of cells with an average width of 2 µm, length of 8–10 µm, and have prominent granules.

Isolate Q2, Lake Qarun (Fig. 2f), has filamentous form. Filaments are thin, straight, or slightly bent, pale to bright blue-green, solitary or entangled, and attenuate at one or both ends. Trichomes are not or are slightly constricted at cell walls. The isolate moves by gliding or pendulum. The filament composes of cylindrical isodiametric cells with 2.5–2.8 µm in width and 2.0–3.0 µm in length. Apical cells are long conical with rounded, slightly hooked, or bent apex and do not capitate. The isolate reproduces by disintegration into small parts, without the formation of necridic cells.

### Molecular identifications based on the 16S rRNA gene and cbbL sequencing

**Fig. 3:**
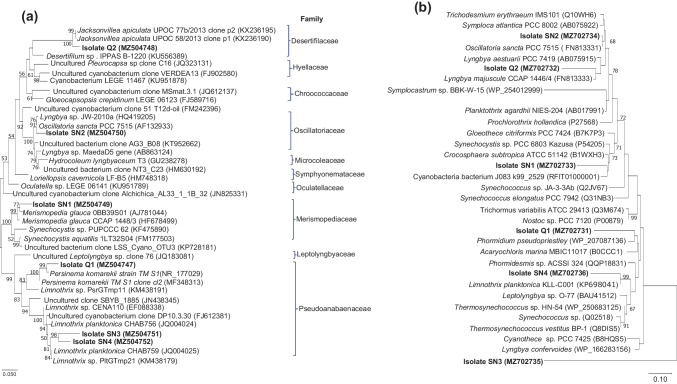
Phylogenetic trees are based on partial 16S rRNA gene nucleotide sequences (**a**) and cbbL deduced amino acid sequences (**b**). Consensus trees were constructed by compatibility of maximum likelihood, neighbor-joining, and maximum-parsimony algorithms. Bootstrap values were calculated from 1000 replicates, and only values more than 50 were specified at the nodes

Isolate SN1 had 16S rRNA gene nucleotide identity percentage of 95.5% with that of *Merismopedia glauca* (acc. no. AJ781044), showing monophyletic and paraphyletic lineages with *M. glauca* isolates (Fig. 3a) (Rajaniemi-Wacklin et al. 2006). The deduced amino acid sequence of the *cbbL* of isolate SN1 had an average identity percentage of 96% with those of *Synechocystis* sp. (P54205), and *Crocosphaera subtropica* (B1WXH3), forming a phylogenetic cluster (Fig. 3b). The 16S rRNA gene nucleotide sequence and *cbbL* deduced amino acid sequence of isolate SN2 showed identities 97.8% and 99.6%, respectively, with those of belonging to *Oscillatoria sancta*, representing similar phylogenetic profiles (Fig. 3a, b). The 16S rRNA gene sequences of isolates SN3 and SN4 formed a clade within the cluster of *Limnothrix* sp*.* (Fig. 3a), showing nucleotide identities, 97.6% and 97.4%, respectively, with those of previously recorded *Limnothrix planctonica* isolates (Zhu et al. 2012). On the other hand, the isolate SN3 represented a unique monophyletic lineage, based on *cbbL* phylogeny (Fig. 3b). The *cbbL* of isolate SN4 formed a monophyletic clade with *Limnothrix planktonica* KLL-C001, with 92.4% amino acid identity (Fig. 3b). Isolate Q1 had 16S rRNA gene nucleotide identity 97.8%, with that of *Persinema komarekii*, where both clustered together in a monophyletic clade (Fig. 3a). The *cbbL* sequence of isolate Q1 was clustered with that of *Phormidium pseudopriestley* (WP_207087136), showing 81.3% amino acid identity (Fig. 3b). The 16S rRNA gene sequence of isolate Q2 was localized in the cluster of *Jacksonvillea apiculate*, representing 96.9% nucleotide identity with those of *Jacksonvillea apiculate* clones (Fig. 3a). The *cbbL* of the isolate Q2 formed a monophyletic clade with *Lyngbya majuscule* (Fig. 3b), and both showed 92.5% amino acid identity.

### Phytochemical constituents

The phytochemical constituents varied from one isolate to another and were significantly different at *p* value < 0.05 (Table 1). The total carbohydrate content varied from 187.0 ± 2.8 to 274 ± 14.5 mg/g DW in isolates SN1 and Q2, respectively. The total protein values ranged from 147.2 ± 7.8 to 364.7 ± 6.4 mg/g DW in isolates SN1 and SN4, respectively (Table 1). The total lipid content fluctuated between 35.5 ± 1.2 mg/g DW in isolate Q2 to 67.6 ± 0.2 mg/g DW in isolate SN4.

**Table 1:** Biochemical constituents of Cyanobacterial isolates

Isolate name	Carbohydrate mg/g DW	Protein mg/g DW	Lipid mg/g DW	AFDW mg/g DW
Isolate SN1	187 ± 8.5	147.2 ± 7.8	48.9 ± 4.4	841.2 ± 4
Isolate SN2	211 ± 10.7	268.5 ± 8.6	57.1 ± 0.8	885.7 ± 2.2
Isolate SN3	187 ± 2.8	215.2 ± 3.7	46.5 ± 1.2	840.6 ± 4
Isolate SN4	266 ± 4.9	364.7 ± 6.4	67.6 ± 0.2	887.1 ± 1.3
Isolate Q1	238 ± 12.0	246.6 ± 2.6	46.8 ± 0.3	824.8 ± 3.2
Isolate Q2	274 ± 14.5	302.8 ± 1.9	35.5 ± 1.2	891.8 ± 2.8

Results were mean ± SD of triplicates expressed as milligram/gram dry weight (mg/g DW)

*AFDW* ash-free dry weight

Total phycobiliproteins (TPBPs) ranged between 82.2 ± 20 and 250 ± 18 mg/g DW for isolates SN4 and SN2, respectively (Table 2). The isolate Q1 had the highest content, 114 ± 20.7 mg/g DW, of phycocyanin (PC), while the isolate SN2 harbored the largest amount of allophycocyanin (APC), 71.0 ± 4.8 mg/g DW, and phycoerythrin (PE) (98 ± 6.7 mg/g DW) (Table 2). The isolate SN4 had a minimal amount of PC (60.9 ± 13.4 mg/g DW), while the isolate SN1 had a minimal amount of APC (17.3 ± 4.6 mg/g DW). The lowest PE content, 1.2 ± 0.1 mg/g DW, was recorded in isolate SN3 (Table 2).

**Table 2:** Pigment contents of Cyanobacterial isolates, normalized to mg/g DW

Isolate name	PC	APC	PE	TPBP	Chl-a	Carotenoids	TPBP/Chl-a	Carot/Chl-a
Isolate SN1	70.2 ± 13.1	17.3 ± 4.6	5.1 ± 0.6	92.7 ± 18	4.9 ± 0.1	0.253 ± 0.01	18.9	0.051
Isolate SN2	81.5 ± 6.2	71 ± 4.8	98 ± 6.7	250 ± 18	3.3 ± 0.5	0.044 ± 0.01	75.8	0.013
Isolate SN3	95.3 ± 1.1	41.5 ± 1.1	1.2 ± 0.1	138 ± 0.1	6.6 ± 0.1	0.15 ± 0.02	20.9	0.023
Isolate SN4	60.9 ± 13.4	18 ± 6.5	3.3 ± 0.4	82.2 ± 20	10 ± 0.6	0.261 ± 0.01	8.2	0.026
Isolate Q1	114 ± 20.7	33.4 ± 3.9	4.7 ± 0.2	152 ± 25	8 ± 0.1	0.067 ± 0.012	19.0	0.008
Isolate Q2	67.5 ± 0.2	38.9 ± 0.7	4.4 ± 0.3	111 ± 0.8	5.2 ± 2.5	0.045 ± 0.004	21.3	0.009

Results were mean ± SD of triplicates expressed as milligram/gram dry weight (mg/g DW)

*PC* phycocyanin, *APC* allophycocyanin, *PE* phycoerythrin, *TPBP* total phycobiliproteins, *Chl-a* chlorophyll-a, *carot.* carotenoids

The current isolates had carotenoid contents with different concentrations, from 0.044 ± 0.01 to 0.261 ± 0.01 mg/g DW for isolates SN2 and SN4, respectively (Table 2). The Chlorophyll-a content varied from 3.3 ± 0.5 mg/g DW in isolate SN2 to 10.0 ± 0.6 mg/g DW in SN4 (Table 2). The ash-free dry weight amount fluctuated between 824.8 ± 3.2 and 891.8 ± 2.8 mg/g DW in isolates Q1 and Q2, respectively (Table 1).

### Discussion

The current study targeted the characterization of Cyanobacterial isolates, which were recovered from topological and physicochemical distinct lakes. The isolate characterization tools included morphology, molecular, and biochemical features. We demonstrated the phylogenetic positions of the current isolates based on two types of marker genes, the 16S rRNA gene, which represents the structural phylogeny marker, and *cbbL*, a representative marker of functional phylogeny for Cyanobacteria. The morphology of the isolate SN1 was similar to that of *Merismopedia glauca* (Ehrenberg) Kützing 1845, a feature supported by the rRNA gene phylogeny profile. Although the family Merismopediaceae harbors about six known genera, only the *cbbL* sequence of the genus *Synechocystis* has been recorded (Kaneko et al. 1996). However, the current *cbbL* sequence was the first record for an isolate belonging to the genus *Merismopedia*, generating *cbbL* data for this genus in lakes. However, the genus *Merismopedia* has been recorded, through a microscopic survey, as a major component of Cyanobacterial blooms in Lake Nasser (Goher et al. 2021).

Both morphology and molecular tools confirmed that the isolate SN2 belonged to the genus *Oscillatoria*, representing a new strain of species *O. sancta* (Gomont 1892). The genus *Oscillatoria* has been reported to be widely spread in the transition and lacustrine zones of Lake Nasser (Salem 2011). *O. sancta* has been found to be frequent in freshwater aquatic bodies that have pH value within the alkaline range and even in the presence of low nutrient content, an optimizing condition for lacustrine zones of Lake Nasser (Halder 2017; Zaher et al. 2021).

Both the morphological criteria and 16S rRNA gene homology confirmed that the isolates SN3 and SN4 belonged to the genus *Limnothrix* (Meffert 1988), but the *cbbL* phylogeny localized SN3 isolate as a unique phylogenetic lineage. The deviation in *cbbL* phylogenetic position of the isolate SN3 may be due to lacking knowledge about the diversity of *cbbL* for the genus *Limnothrix*. In terms of growth, isolate SN3 formed planktonic growth through the culture vessels, while isolate SN4 formed mat settled at the walls and bottom of the culture flask. In addition, both isolates, SN3 and SN4, showed differences in the phytochemical contents, suggesting differentiated isolates*.* A polyphasic study on *Limnothrix planktonica* strains, isolated from shallow eutrophic lakes in China, has suggested that *Limnothrix* species are polyphyletic and their taxonomy requires further examination (Zhu et al. 2012). *Limnothrix planktonica* has been reported to be the most abundant Cyanobacterial species in eutrophic European lakes (Noges et al. 2008). These observations may suggest that the current *Limnothrix*-like isolates are bioindicators of eutrophication in Lake Nasser.

The phylogenies of some isolates based on *cbbL* differed from those based on the 16S rRNA gene. The phylogenetic profile derived from *cbbL* sequences sometimes shows a relationship between distant taxonomic species, which are classified based on rRNA gene phylogeny (Iniguez et al. 2020). This disagreement between the 16S rRNA gene and *cbbL* phylogenies may refer to the concept of the possibility of horizontal gene transfer of *cbbL* in the evolution of Cyanobacteria (Delwiche and Palmer 1996; Badger et al. 2002; Iniguez et al. 2020). This rampant horizontal *cbbL* transfer, in addition to lacking knowledge of *cbbL* sequence for some Cyanobacterial genera, may explain the variation in the phylogenetic position of isolates, Q1 and Q2, in both current trees*.* The isolate Q1 had morphological features and rRNA gene homology similar to that of *Persinema komarekii*, a species, which commonly occurs in the highly radiated aquatic area (Heidari et al. 2018). However, some areas of Lake Qarun are characterized by the existence of radioactive ^40^ K due to excessive discharge of K-containing fertilizers, from agricultural drains into the lake (Imam 2005; Darwish et al. 2013; Amin 2015). These environmental conditions may favor the occurrence of *Persinema*-like isolate Q1. Both of morphology and rRNA gene homology of isolate Q2 showed similarity with *Jacksonvillea apiculata*, a species that belongs to Desertifilaceae (Hasler et al. 2017). Both *Persinema komarekii* and *Jacksonvillea apiculata* have limited studies and have no *cbbL* sequences deposited in the DNA database. However, the taxonomic observation of isolates Q1 and Q2 may support the concept that functional *cbbL* phylogeny shows conservation among distant Cyanobacterial taxa (Liu et al. 2017). The *cbbL* phylogenetic radiation within Cyanobacteria may be due to a response to environmental stresses (Jaffe et al. 2018).

The carbohydrate contents of isolates SN4, Q1, and Q2 (Table 1) were higher than those recorded in members of the closest genera *Synechococcus* (148 mg/g DW), *Oscillatoria* (186 mg/g DW), and *Lyngbya* (173 mg/g DW), besides the species, *Arthrospira platensis* (146.7 mg/g DW), *Anabaena* sp. (114.6 mg/g DW), *Merismopedia tenussima* (109.3 mg/g DW), and *Spirulina platenesis* (50.7 mg/g DW) (Patel et al. 2017; Cheng et al. 2019; Issa et al. 2020; Ennaji et al. 2021), suggesting suitable sources for carbohydrate-dependent industry, such as bioethanol production. On the other hand, the protein contents of the isolates, SN4, Q1, and Q2 (Table 1), were higher than those have been recorded in *Anabaena* sp. (118.5 mg/g DW), *Merismopedia tenussima* (72.3 mg/g DW), *Spirulina platenesis* (160.7 mg/g DW) (Issa et al. 2020), and *Nostoc* sp. (109.8–280.2 mg/g DW), and other seven Cyanobacterial species, *Oscillatoria foreaui*, *O*. *calcuttensis*, *O. acuminate*, *Gloeocapsa livida*, *Lyngbya limnetica*, *Calothrix fusca*, and *Scytonema bohneri*, which have protein contents fluctuated between 16 and 70 mg/g DW (Rajeshwari and Rajashekhar 2011). The protein contents obtained from the current isolates were similar to those recorded from *Nostoc* sp. (109.8–280.2 mg/g DW) and *Cylindrospermum* sp. (141.1–366.9 mg/g DW) (Borah et al. 2016). So, the current study isolates could be used as a protein source or as food additives in the food industry.

The lipid contents of current isolates were consistent with those previously recorded in Cyanobacterial species *Spirulina platensis*, *Oscillatoria acuta*, *Calothrix* sp., *Lyngbya* sp. *Leptolyngbya* sp., *Synechococcus* sp., *Nostoc muscorum*, *Oscillatoria marina*, *Anabaena* sp. *Cyanobium* sp., *Limnothrix* sp., *Nostoc* sp., and *Merismopedia tenuissima* that had lipid content fluctuated between 25 and 66 mg/g DW (Sahu et al. 2013; Oliveira et al. 2018; Issa et al. 2020). However, further studies should be carried out for optimizing the growth parameters to increase the lipid content of these isolates to be more attractive for commercial production (Vargas et al. 1998).

Phycobiliproteins (PBPs), phycocyanin, allophycocyanin, and phycoerythrin are light-harvesting colored proteins produced by Cyanobacteria, as photosynthetic accessory pigments and have biotechnological potentials. The phycobiliprotein compositions in current Cyanobacterial isolates showed variations. Isolate Q1 was attractable for its highest phycocyanin content and can be used as an alternative for *Arthrospira platensis* (Basheva et al. 2018) since isolate Q1 was recovered from heavily polluted marine water. Isolate SN2 can be added to *Oscillatoria* members for mass production of phycoerythrin (Rai and Rajashekhar 2015). In their study of phycobiliproteins production by 18 Cyanobacterial strains, Hemlata and Fatma (2009) recorded the highest amount of phycobiliproteins, 91 mg/g DW, in *Anabaena* NCCU-9. However, this amount was lower than those recorded in current isolates. Chlorophyll-a along with phycobiliproteins is the main photosynthetic pigment in Cyanobacteria (Munir et al. 2013). The chlorophyll-a and carotenoid contents of isolate SN4 were higher than those recorded for *Synechococcus elongates*, *S. aeruginosus*, and *Phormidium fragile* (Jeevanantham et al. 2019). Generally, isolates SN2 and Q1 could be sources for pigment production.

### Conclusion

The study aimed to characterize Cyanobacterial isolates from different desert lakes and to assess their biochemical composition with a view toward biotechnology applications. The study recorded new six Cyanobacterial isolates, from Egyptian lakes. Isolates were characterized based on morphology, genetic signatures, and biochemical composition. The isolates SN1, SN2, SN3, SN4, Q1, and Q2 belonged to the genera, *Merismopedia*, *Oscillatoria*, *Limnothrix*, *Persinema*, and *Jacksonvillea*, respectively, according to phylogenetic analyses of the 16S rRNA gene. In addition, the study presented the first *cbbL* sequence for the genera *Merismopedia*, *Persinema*, and *Jacksonvillea*, represented by the isolates SN1, Q1, and Q2. On the other hand, isolates, SN4 and Q2 had the highest protein and carbohydrate contents, respectively, while isolate SN2 showed contents rich with pigments, and may therefore have biotechnological potential.

## Data Availability

Data are available from the authors upon request.

## References

[CR1] Abd El-Aal RF, El Sayed SM, Attia MS, Donia NS, Goher ME (2020). Pollution indices and distribution pattern of heavy metals in Lake Qarun water. Egypt EJABF.

[CR2] Abdel-Gawad SS, Abdel-Aal EI (2018). Impact of flood cycle on phytoplankton and macro invertebrates associated with Myriophyllum spicatum in Lake Nasser Khors (Egypt). J Biol Sci.

[CR3] Abd El-Karim MS (2012). Present status and long term changes of phytoplankton in closed saline basin with special reference to the effect of salinity. Int j Environ.

[CR4] Allen MM (1968). Simple conditions for growth of unicellular blue-green algae. J Gen Microbiol.

[CR5] Andersen RA, Kawachi M, Andersen RA (2005). Traditional microalgae isolation techniques. Algal Culturing Techniques.

[CR6] Amin RM (2015). Radioactivity Levels in Some Sediments and Water Samples from Qarun Lake by Low-Level Gamma Spectrometry. IJSR.

[CR7] AOAC (2000) Official method of analysis of AOAC International, 17th edn. Horwitz W (ed). AOAC International, Maryland, USA

[CR8] Badger MR, Hanson D, Price GD (2002). Evolution and diversity of CO_2_ concentrating mechanisms in cyanobacteria. Funct Plant Biol.

[CR9] Basheva D, Moten D, Stoyanov P, Belkinova D, Mladenov R, Teneva I (2018). Content of phycoerythrin, phycocyanin, alophycocyanin and phycoerythrocyanin in some cyanobacterial strains: applications. Eng Life Sci.

[CR10] Bennett A, Bogorad L (1973). Complementary chromatic adaptation in a filamentous blue-green alga. J Cell Biol.

[CR11] Borah D, Vimala N, Thajuddin N (2016). Biochemical composition and chemotaxonomy of cyanobacteria isolated from Assam. North-East India Phykos.

[CR12] Castenholz RW (1989) Subsection IV. Order Nostocales. In: Staley JT, Bryant MP, Pfennig N, Holt JG (eds) Bergey’s Manual of Systematic Bacteriology, vol 3. Williams & Wilkins, Baltimore, pp 1780–1793

[CR13] Castenholz RW, Wilmotte A, Herdman M, Rippka R, Waterbury JB, Iteman I, Hoffmann L (2001) Phylum BX. Cyanobacteria. Bergey’s Manual^®^ of Systematic Bacteriology. Springer, New York, NY, pp 473–599. 10.1007/978-0-387-21609-6_27

[CR14] Cheng J, Yue L, Ding L, Li YY, Ye Q, Zhou J, Lin R (2019). Improving fermentative hydrogen and methane production from an algal bloom through hydrothermal/steam acid pretreatment. Int J Hydrog Energy.

[CR15] Choi HJ, Joo J, Kim J, Wang P, Ki J, Han M (2018) Morphological characterization and molecular phylogenetic analysis of Dolichospermum hangangense (Nostocales, Cyanobacteria) sp. nov. from Han River, Korea. Algae 33:143–156. 10.4490/algae.2018.33.5.2

[CR16] Dadheech PK, Krienitz L, Kotut K, Ballot A, Casper P (2009). Molecular detection of uncultured cyanobacteria and aminotransferase domains for cyanotoxin production in sediments of different Kenyan lakes. FEMS Microbiol Ecol.

[CR17] Darwish SM, El-Bahi SM, Sroor AT, Arhoma NF (2013). Natural radioactivity assessment and radiological hazards in soils from Qarun Lake and Wadi El Rayan in Faiyum. Egypt Open J Soil Sci.

[CR18] Delwiche CF, Palmer JD (1996). Rampant horizontal transfer and duplication of RuBisCO genes in eubacteria and plastids. Mol Biol Evol.

[CR19] Demay J, Bernard C, Reinhardt A, Marie B (2019). Natural products from cyanobacteria: focus on beneficial activities. Mar Drugs.

[CR20] Dvorak P, Poulıckova A, Hasler P, Belli M, Casamatta DA, Papini A (2015). Species concepts and speciation factors in cyanobacteria, with connection to the problems of diversity and classification. Biodivers Conserv.

[CR21] Dvorak P, Hindak F, Hasler P, Hindakova A, Poulıckova A (2014) Morphological and molecular studies of Neosynechococcus sphagnicola, gen. et sp. nov. (Cyanobacteria, Synechococcales). Phytotaxa 170:24–34. 10.11646/phytotaxa.170.1.3

[CR22] Elsaied HE (2007) Molecular genetic monitoring of bacterial communities in Manzala lake, Egypt, based on 16S rRNA gene analysis. Egypt J Aquat Res 33:179–194. http://hdl.handle.net/1834/2206

[CR23] Elsaied H, Naganuma T (2001). Phylogenetic diversity of ribulose1,5-bisphosphate carboxylase/oxygenase large-subunit genes from deep-sea microorganisms. Appl Environ Microbiol.

[CR24] Elsaied H, Kimura H, Naganuma T (2002). Molecular characterization and endosymbiotic localization of the gene encoding ribulose 1,5-bisphosphate carboxylase-oxygenase (RuBisCO) form II in the deep-sea vestimentiferan trophosome. Microbiology.

[CR25] Elsaied HE, Soliman T, Abu-Taleb HT, Goto H, Jenke-Kodam H (2019). Phylogenetic characterization of eukaryotic and prokaryotic gut flora of Nile tilapia, Oreochromis niloticus, along niches of Lake Nasser, Egypt, based on rRNA gene high-throughput sequences. Ecol Genet Genom.

[CR26] Ennaji H, Bourhia M, Taouam I, Falaq A, Bellahcen TO, Salamatullah AM, Alzahrani A, Alyahya HK, Ullah R, Ibenmoussa S, Khlil N, Cherki M (2021). Physicochemical evaluation of edible cyanobacterium Arthrospira platensis collected from the South Atlantic Coast of Morocco: a promising source of dietary supplements. Evid Based Complement Alternat Med.

[CR27] Fathi AA, Flower RJ (2005). Water quality and phytoplankton communities in Lake Qarun (Egypt). Aquat Sci.

[CR28] Flefil NS, Mahmoud AMA (2021) The seasonal fluctuations of phytoplankton diversity and its biochemical components in Lake Qarun, Egypt. EJABF 25:131–145. 10.21608/EJABF.2021.198132

[CR29] Folch J, Lees M, Stanley GHS (1957). A simple method for the isolation and purification of total lipids from animal tissues. J Biol Chem.

[CR30] Goher ME, Napiórkowska-Krzebietke A, Aly W, El-Sayed SM, Tahoun UM, Fetouh MA, Hegab MH, Haroon AM, Sabae SA, Abdel-Aal EI, Nassif MG, Hussian AM (2021). Comprehensive insight into Lake Nasser environment: water quality and biotic communities: a case study before operating the Renaissance dam. Water.

[CR31] Gomont M (1892) Monographie des Oscillariees (Nostocacees Homocyst6es). Ann Sci Nat ser. 7, Bot. 15:263–368; 16:91–264

[CR32] Goris J, Klappenbach JA, Vandamme P, Coenye T, Konstantinidis KT, Tiedje JM (2007). DNA–DNA hybridization values and their relationship to whole-genome sequence similarities. Int J Syst Evol Microbiol.

[CR33] Guillard RRL, Andersen RA (2005). Purification methods for microalgae. Algal Culturing Techniques.

[CR34] Guiry MD, Guiry GM (2021) AlgaeBase. World-wide electronic publication, National University of Ireland, Galway. https://www.algaebase.org

[CR35] Halder N (2017) Taxonomy and biodiversity of the genus Oscillatoria Vauch. ex Gom. (Cyanoprokaryota: Oscillatoriales) with ecological notes from Hooghly in West Bengal, India. Braz J Biol Sci 4:89–101. 10.21472/bjbs.040710

[CR36] Hasler P, Casametta D, Dvorák P, Poulícová A (2017) Jacksonvillea apiculata (Oscillatoriales, Cyanobacteria) gen. & sp. nov.: a new genus of filamentous, epipsamic Cyanobacteria from North Florida. Phycologia 56(3):284–295. 10.2216/16.62.1

[CR37] Heidari F, Hauer T, Zima JR, Riahi H (2018) New simple trichal Cyanobacterial taxa isolated from radioactive thermal springs. Fottea 18(2):137–149. 10.5507/fot.2017.024

[CR38] Hemlata FT (2009). Screening of cyanobacteria for phycobiliproteins and effect of different environmental stress on its yield. Bull Environ Contam Toxicol.

[CR39] Imam N, El-Sayed SM, Goher ME (2020). Risk assessments and spatial distributions of natural radioactivity and heavy metals in Nasser Lake. Egypt Environ Sci Pollut Res.

[CR40] Imam NAM (2005) Physical characteristics and evaluation of natural radioactivity in Lake Qarun, Egypt. Master thesis (Nuclear physics), Ain Shams Univ. Egypt

[CR41] Iniguez C, Capo-Bauc S, Niinemets U, Stoll H, Aguilo-Nicolau P, Galmes J (2020). Evolutionary trends in RuBisCO kinetics and their co-evolution with CO_2_ concentrating mechanisms. TPJ.

[CR42] Issa A, Ali E, Abdel-Basset R, Hassan S, Awad M, Ebied A (2020). Application of three Cyanobacteria in foods and feeds biotechnology: phosphorus affects. Pak J Biol Sci.

[CR43] Jaffe AL, Castelle CJ, Dupont CL, Banfield JF (2018). Lateral gene transfer shapes the distribution of RuBisCO among candidate phyla radiation bacteria and DPANN archaea. Mol Biol Evol.

[CR44] Jeevanantham G, Vinoth M, Hussain JM, Muruganantham P, Ahamed AK (2019). Biochemical characterization of five marine cyanobacteria species for their biotechnological applications. J Pharmacogn Phytochem.

[CR45] Kaneko T, Sato S, Kotani H (1996). Sequence analysis of the genome of the unicellular cyanobacterium Synechocystis sp. strain PCC6803. II. Sequence determination of the entire genome and assignment of potential protein-coding regions. DNA Res.

[CR46] Kim S, Kim D, Cho SW, Kim J, Kim JS (2014). Highly efficient RNA-guided genome editing in human cells via delivery of purified Cas9 ribonucleoproteins. Genome Res.

[CR47] Komárek J, Anagnostidis K (2005) Cyanoprokaryota 2 Teil: Oscillatoriales. In: Büdel B, Gärtner G, Krienitz L, Schagerl M (eds) Süßwasserflora von Mitteleuropa, Bd. 19 (2), Elsevier GmbH, München, pp 1–759

[CR48] Komárek J, Kaštovský J, Mareš J, Johansen JR (2014). Taxonomic classification of cyanoprokaryotes (cyanobacterial genera) 2014, using a polyphasic approach. Preslia.

[CR49] Konstantinidis KT, Ramette A, Tiedje JM (2006). The bacterial species definition in the genomic era. Philos Trans R Soc Lond B Biol Sci.

[CR50] Kützing FT (1845) Phycologia germanica, d. i. Deutschlands Algen in bündigen Beschreibungen. Nebst einer Anleitung zum Untersuchen und Bestimmen dieser Gewächse für Anfänger. pp. [i]-x, [1]-340 ['240']. Nordhausen: zu finden bei Wilh. Köhne

[CR51] Lane DJ, Stackebrandt E, Goodfellow M (1991). 16S/23S rRNA sequencing. Nucleic acid techniques in bacterial systematics.

[CR52] Lorenzen CJ (1967). Determination of chlorophyll and pheopigments: spectrophotometric equations. Limnol Oceanogr.

[CR53] Lowry OH, Rosenbrough NJ, Farr AL, Randall RJ (1951). Protein measurement with the Folin – phenol reagent. J Biol Chem.

[CR54] Liu D, Chettiyan R, Ramya S, Mueller-cajar O (2017). Surveying the expanding prokaryotic RuBisCO multiverse. FEMS Microbiol Lett.

[CR55] Meffert ME (1988). Limnothrix Meffert nov. gen. - the unsheathed planktic cyanophycean filaments with polar and central gas vacuoles. Arch Hydrobiol.

[CR56] Mesbah NM, Hedrick DB, Peacock AD, Rohde M, Wiegel J (2007). Natranaerobius thermophilus gen. nov., sp. nov., a halophilic, alkalithermophilic bacterium from soda lakes of the Wadi An Natrun, Egypt, and proposal of Natranaerobiaceae fam. nov. and Natranaerobiales ord. nov. Int J Syst Evol Microbiol.

[CR57] Miller SR, Purugganan MD, Curtis SE (2006). Molecular population genetics and phenotypic diversification of two populations of the thermophilic Cyanobacterium Mastigocladus laminosus. Appl Environ Microbiol.

[CR58] Munir N, Sharif N, Naz S, Manzoor F (2013). Algae: a potent antioxidant source. Sky J Microbiol Res.

[CR59] Nagarajan M, Maruthanayagam V, Sundararaman M (2012). A review of pharmacological and toxicological potentials of marine cyanobacterial metabolites. J Appl Toxicol.

[CR60] Noges T, Laugaste R, Nõges P, Tõnno I (2008). Critical N: P ratio for cyanobacteria and N2-fixing species in the large shallow temperate lakes Peipsi and Võrtsjärv, North-East Europe. Hydrobiologia.

[CR61] Oliveira DT, Vasconcelos CT, Feitosa AMT, Aboim JB, Oliveira AN, Xavier LP, Santos AS, Gonçalves EC, Filho GNR, Nascimento LAS (2018). Lipid profile analysis of three new Amazonian cyanobacteria as potential sources of biodiesel. Fuel.

[CR62] Patel VK, Sundaram S, Patel AK, Kalra A (2017). Characterization of seven species of Cyanobacteria for high-quality biomass production. Arab J Sci Eng.

[CR63] Quero-Jiménez PC, Montenegro ON, Sosa R, De la Torre JB, Valero Acosta J, Pérez LD, Rodríguez AS, Méndez RR, Alonso AC, Corrales AJ, Hernández NB (2019) Total carbohydrates concentration evaluation in products of microbial origin. Afinidad LXXVI 587:195–203. https://raco.cat/index.php/afinidad/article/view/361475

[CR64] Rai SV, Rajashekhar M (2015) Phytochemical screening of twelve species of phytoplankton isolated from Arabian Sea coast. J Coast Life Med 3:857–863. 10.12980/jclm.3.2015j5-83

[CR65] Rajaniemi-Wacklin P, Rantala A, Mugnai M, Turicchia S, Ventura S, Komárková J, Lepistö L, Sivonen K (2006). Correspondence between phylogeny and morphology of Snowella spp. and Woronichinia naegeliana, cyanobacteria commonly occurring in lakes. J Phycol.

[CR66] Rajeshwari KR, Rajashekhar M (2011). Biochemical composition of seven species of Cyanobacteria isolated from different aquatic habitats of western ghats, Southern India. Braz Arch Biol Technol.

[CR67] Redwan M, Elhaddad E (2017). Heavy metals seasonal variability and distribution in Lake Qarun sediments, El-Fayoum. Egypt J Afr Earth Sci.

[CR68] Rippka R, Deruelles J, Waterbury JB, Herdman M, Stanier RY (1979). Generic assignments, strain histories and properties of pure cultures of Cyanobacteria. J Gen Microbiol.

[CR69] Ritchie RJ (2006). Consistent sets of spectrophotometric chlorophyll equations for acetone, methanol and ethanol solvents. Photosynth Res.

[CR70] Sahu A, Pancha I, Jain D, Paliwal C, Ghosh T, Patidar S, Bhattacharya S, Mishra S (2013). Fatty acids as biomarkers of microalgae. Phytochem.

[CR71] Salem TA (2011). Variation of water quality and phytoplankton along different zones of Aswan High Dam Reservoir. EJABF.

[CR72] Shen M, Li Q, Ren M, Lin Y, Wang J, Chen L, Li T, Zhao J (2019) Trophic status is associated with community structure and metabolic potential of planktonic microbiota in plateau lakes. Front Microbiol 10:2560. 10.3389/fmicb.2019.02560 10.3389/fmicb.2019.02560 PMC6853845 31787952

[CR73] Singh SK, Major SR, Cai H, Chen F, Hill RT, Li Y (2018). Draft genome sequences of Cloacibacterium normanense IMET F, a microalgal growth-promoting bacterium, and Aeromonas jandaei IMET J, a microalgal growth-inhibiting bacterium. Genome Announc.

[CR74] Tamura K, Stecher G, Kumar S (2021). MEGA11: Molecular evolutionary genetics analysis Version 11. Mol Biol Evol.

[CR75] Vargas MA, Rodríguez H, Moreno J, Olivares H, Campo JAD, Rivas J, Guerrero MG (1998). Biochemical composition and fatty acid content of filamentous nitrogen-fixing Cyanobacteria. J Phycol.

[CR76] Walter JM, Coutinho FH, Dutilh BE, Swings J, Thompson FL, Thompson CC (2017). Ecogenomics and taxonomy of Cyanobacteria phylum. Front Microbiol.

[CR77] Wellburn AR (1994). The spectral determination of chlorophylls a and b, as well as total carotenoids, using various solvents with spectrophotometers of different resolution. J Plant Physiol.

[CR78] Whitton BA, Potts M (eds) (2000) The ecology of Cyanobacteria Kluwer Academic Publishers, Dordrecht, Netherlands. 10.1007/0-306-46855-7_1

[CR79] Wilmotte A, Dail Laughinghouse IVH, Capelli C, Rippka R, Salmaso N, Kurmayer R (2017). Taxonomic identification of Cyanobacteria by a polyphasic approach. Molecular tools for the detection and quantification of toxigenic Cyanobacteria.

[CR80] Zaher SS, Abd El-Hady HH, Khalifa N (2021) Phytoplankton composition and its biochemical contents in a subtropical reservoir (Lake Nasser, Egypt) during flood season. EJABF 25:443–459. 10.21608/EJABF.2021.144397

[CR81] Zaher SS, Ibrahim EA (2018) Phytoplankton blooming in Lake Qarun in relation to Chlorophyll-a measured by fluorometric and spectrophotometric techniques. EJABF 22:275–286. 10.21608/EJABF.2018.22002

[CR82] Zaki MA, Ashour M, Heneash AMM, Mabrouk MM, Alprol AE, Khairy HM, Nour AM, Mansour AT, Hassanien HA, Gaber A, Elshobary ME (2021). Potential applications of native cyanobacterium isolate (Arthrospira platensis NIOF17/003) for biodiesel production and utilization of its byproduct in marine rotifer (Brachionus plicatilis) production. Sustainability.

[CR83] Zavřel T, Sinetova MA, Červený J (2015) Measurement of chlorophyll-a and carotenoids concentration in cyanobacteria. Bio-protocol 5:e1467 10.21769/bioprotoc.1467

[CR84] Zhu M, Yu G, Li X, Tan W, Renhui L (2012). Taxonomic and phylogenetic evaluation of Limnothrix strains (Oscillatoriales, Cyanobacteria) by adding Limnothrix planktonica strains isolated from central China. Hydrobiologia.

